# Neurobiological intersections of stress and substance use disorders

**DOI:** 10.3389/fnins.2025.1548372

**Published:** 2025-05-01

**Authors:** Vitor Augusto Laurino Juliano, Kairo Alan Albernaz-Mariano, Luiza Helena Halas Covre, Paloma Marinho Jucá, Robbert Mota Pereira, Amadeu Shigeo-de-Almeida, Lucas Luzia Sampaio, Erica de Almeida Duque, Carolina Demarchi Munhoz

**Affiliations:** Department of Pharmacology, Institute of Biomedical Sciences, University of São Paulo, São Paulo, SP, Brazil

**Keywords:** HPA axis, neuroinflammation, mood disorders, early life stress, substance use disorder

## Abstract

Substance use has been intertwined with human history for millennia. Throughout the ages, people have consumed various substances for medicinal, spiritual, and recreational reasons, although occasional use differs significantly from substance use disorders (SUDs). Exposure to lifetime stressors constitutes a significant risk factor for both psychiatric disorders and SUD development and relapse. Indeed, hypothalamic–pituitary–adrenal (HPA) axis modulation, alterations in neuroanatomical and neurotransmitter systems, as well as neuroinflammation are common features of stress-related mood disorders and SUDs. In this mini-review, we will explore how stress exposure influences the SUDs' neurobiological basis on different scales—from large neural circuitries to specific molecular mechanisms—and discuss novel targets for potential treatments.

## 1 Introduction

SUDs are defined as brain diseases characterized by compulsion for drug seeking and intake despite severe negative consequences related to the loss of control and emergence of a negative emotional state (Liu and Li, 2018). According to the 5th Edition of the Diagnostic and Statistical Manual of Mental Disorders (American Psychiatric Association, 2013), SUDs can be classified as mild, moderate or severe. The recently published data from The World Health Organization and the United Nations Office on Drugs and Crime showed that 64 million people worldwide were suffering from SUDs in 2022, which accounts for an increase of 3% over 5 years (Drugs and Crime, 2024), while the global prevalence of mental disorders was 13.0% (Castaldelli-Maia and Bhugra, 2022). Interestingly, the SUD prevalence among individuals with major depressive disorder was 25% (Hunt et al., 2020) and 33% among people with bipolar disorder (Hunt et al., 2016). Also, there is strong evidence of comorbidity of SUD with generalized anxiety disorder (Alegria et al., 2010) and posttraumatic stress disorder (PTSD; McCauley et al., 2012).

Stress is a natural and adaptive response required to sustain life that can be interpreted as any stimulus that changes physiological and/or psychological states (Schneiderman et al., 2005; Le Moal, 2007). Neurons located in the dorsomedial parvocellular subdivision of the paraventricular nucleus of the hypothalamus release corticotropin-releasing factor (CRF) in the hypophyseal portal system in response to stressors, which binds to CRH receptor type 1 (CRHR1) in hypophysis and leads to adrenocorticotropic hormone secretion in the systemic circulation, culminating in glucocorticoids (GCs) release [cortisol in humans and corticosterone (CORT) in rodents]. The GC hormones have genomic (slow) and non-genomic (fast) actions through the mineralocorticoid (MR) or glucocorticoid (GR) receptors. The cytosolic GC-MR/GR complex translocates to the cell nucleus and modulates gene expression by binding to the DNA's glucocorticoid-responsive element (GRE) regions (Beato and Sanchez-Pacheco, 1996) for long-lasting genomic effects. The CORT acts through classical MR and GR inserted in or attached to the plasma membrane for rapid non-genomic action, facilitating or inhibiting ion channels, receptors, and neurotransmitter signaling (Groeneweg et al., 2011). As a crucial stress mediator, GCs play an important role in arousal, cognition, mood, immunity, inflammatory reactions (Oster et al., 2017), and SUD (Mantsch and Gasser, 2015). According to allostasis, depending on the stress nature, intensity, and chronicity, the energy demand may be higher than the organism's resource (allostatic overload), leading to maladaptive responses (McEwen and Wingfield, 2003). Indeed, stress can be a significant risk factor for the development of both psychiatric disorders and SUDs (McGrath and Briand, 2019).

Some limbic regions, such as the ventral tegmental area (VTA), nucleus accumbens (NAc), prefrontal cortex (PFC), amygdala, and bed nucleus of the stria terminalis (BNST), are crucial for governing stress response and different drug use stages. For example, the VTA dopaminergic neurons release dopamine (DA) to other regions responsible for reward processing, such as the NAc and the PFC (Kielbinski et al., 2019). In contrast, the VTA inhibitory interneurons mediate reward-seeking reduction via NAc communication in stressed animals (Lowes et al., 2021). The amygdala is involved in emotional processing, highly responsive to stressors, and strongly related to the withdrawal period, playing a significant role in symptoms such as anxiety, irritability, and unease symptoms present in patients experiencing withdrawal (Stamatakis et al., 2014; Gilpin et al., 2015). More recent data showed that stress disruption of reward responses depends on the amygdala-NAc pathway (Madur et al., 2023). Indeed, the SUD implications in reward and stress (“anti-reward”) systems have long been stated (Volkow et al., 2016).

Given the association between stress and SUD (Nikbakhtzadeh et al., 2023), it is fundamental to clarify what is currently known about the cellular, molecular, and genetic mechanisms governing the relationship between stress and drug use responses to identify new therapeutic targets. We will first address the shared anatomical and neuroendocrine basis of SUD and stress. Then, despite several research models of stress that differ in neurobiological and behavioral effects from each other, we will give a special focus to early life stress (ELS) and cellular stress (i.e., oxidative stress and neuroinflammation) on SUD. Finally, we will explore genetic hallmarks of stress and HPA-axis regulation related to SUD.

### 1.1 Anatomical and neuroendocrine features of stress and SUD

**Figure 1:**
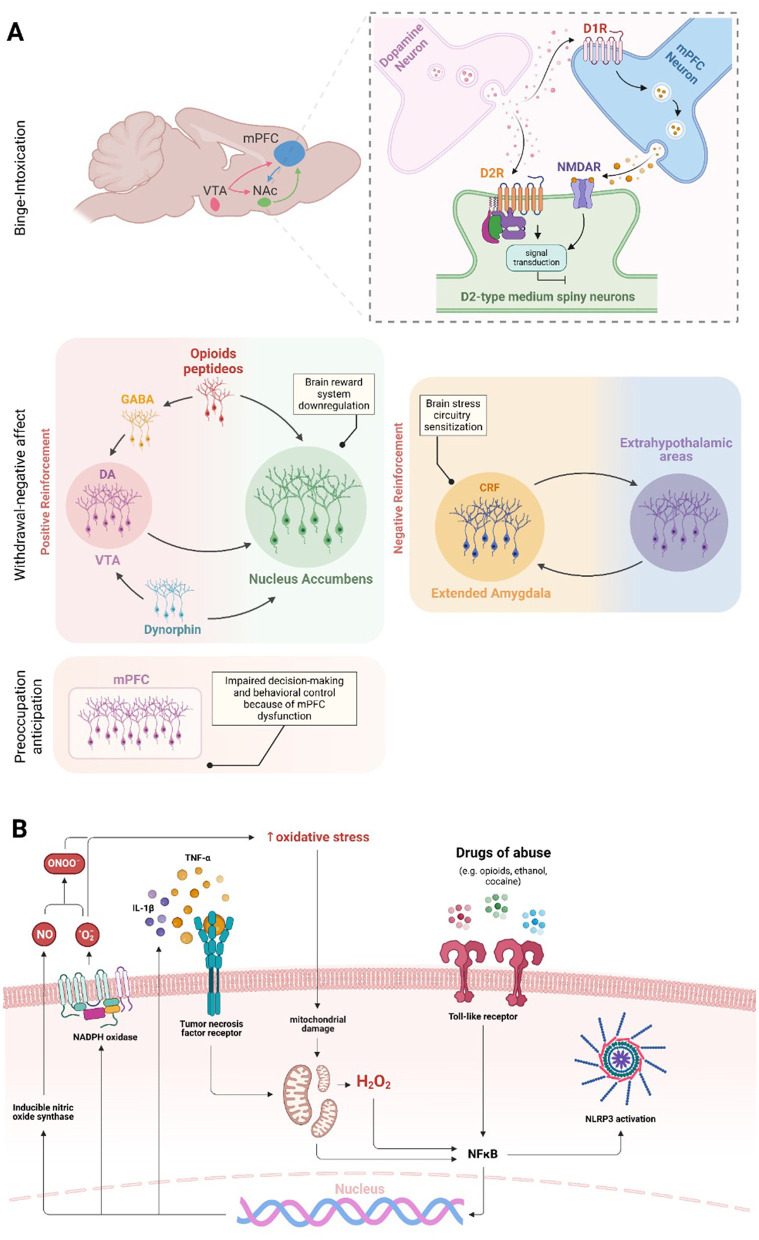
Brain regions and molecular effects involved in substance use and addiction. The three main domains of addiction neurocircuitry correspond to distinct functional areas, including binge/intoxication, associated with reward and incentive salience (activation of D1 dopamine receptor in the VTA-NAc pathway and D2 receptor inhibition in the striatum-cortex pathway—NMDAR are also involved in D2R response); withdrawal/negative affect, linked to negative emotional states and stress (brain reward systems downregulation and stress circuitry sensitization—dopaminergic, CRF, opioid, GABA, and dynorphin systems are involved); and preoccupation/anticipation, related to craving, impulsivity, and executive function; decision making and behavioral control are impaired in consequence of, but not restricted to, mPFC dysfunction **(A)**. Drugs of abuse increase oxidative stress levels in the brain, initiating a continuous cycle that sustains neuroinflammation. The oxidative stress caused by substance use can compromise mitochondrial function, resulting in increased generation of free radicals. Increased oxidative stress contributes to the nuclear translocation and activation of NF-κB in microglial cells and induces the NLRP3 inflammasome activation. In addition, the drug use activates TLR4, which also triggers the activation of microglial NF-κB. Once in the nucleus, NF-κB promotes the increased expression of NOX and iNOS enzymes and pro-inflammatory cytokines, such as TNF-α and IL-1β. The increase in oxidative stress, pro-inflammatory cytokines and NLRP3 ultimately intensifies microglial and astrocytic activation, leading to a cycle of inflammation and oxidative stress in the brain **(B)**.

A three-stage model—including binge/intoxication, withdrawal/negative effects, and preoccupation/anticipation—has been used to explain the transition from drug use to SUD (Koob and Volkow, 2010; Figure 1A). The drug-induced activation of the D1 dopamine receptor in the mesolimbic pathway (from VTA to NAc) and inhibition of D2 receptors in the striatocortical pathway (from the cerebral cortex to striatum) are classically associated with reinforcing, positive drug effects present during *binge stage*—even though μ-opioid receptors and endocannabinoid systems are also involved (Volkow and Morales, 2015). However, sustained drug intake leads to a dynamic readjustment of physiological parameters, including long-term brain changes that result in increased SUD risk and relapse. This process is referred to as the allostatic theory of addiction (Koob and Le Moal, 1997, 2001), which ultimately leads to *withdrawal/negative* effects. Indeed, the increase in reward threshold due to dopaminergic system downregulation is an early hallmark of drug-induced neuroadaptations, leading to the deficit in natural reward experience called anhedonia (Volkow et al., 2009). In addition to the dopaminergic system, CRF, dynorphin, and hypocretin are also modulated by chronic drug intake and related to the *withdrawal/negative* feelings stage. The CRF system is responsible for HPA-axis dysregulation followed by alterations in the extended amygdala, an extra-hypothalamic area composed of the central amygdala (CeA), BNST, and a transition zone in the posterior part of the medial NAc (Koob, 2008). The dynorphin-κ opioid system also modulates the extended amygdala. At the same time, hypocretin (derived exclusively from the lateral hypothalamus) interacts with noradrenergic, cholinergic, serotonergic, histaminergic, and dopaminergic systems, in addition to its role in HPA axis regulation (Koob, 2008). Regarding the *preoccupation/anticipation stage*, prefrontal cortex (PFC) dysfunction has been associated with the loss of control and compulsive drug-taking characteristic of this stage because of its role in decision-making and self-regulation (Figure 1A). Transcranial direct current stimulation (tDCS) over the dorsolateral prefrontal cortex (DLPFC) reduced craving immediately after the session and 1 month later in individuals with methamphetamine-use disorder (Alizadehgoradel et al., 2020). Moreover, individuals with SUD showed decreased left dorsal anterior cingulate cortex (dACC) and right middle frontal gyrus (MFG) activation compared to healthy controls (Le et al., 2021).

Notably, the neuroanatomical and neurotransmitter systems governing SUD substantially overlap with stress response. In this regard, ethanol intake was prevented by prior GR (but not MR) antagonism (Koenig and Olive, 2004). Both stress and GCs increase DA synthesis (Baik, 2020) and reduce its clearance (Parnaudeau et al., 2014), which influences the sensitization to psychomotor stimulants, increases substance-induced conditioned place preference and self-administration of cocaine, amphetamine, heroin, and relapse to cocaine seeking (Yap and Miczek, 2008). Given that contextual memory retrieval depends on the hippocampal GR (Roozendaal et al., 2003), it could be part of the mechanism governing the intense craving and anxiety reported by SUD patients in response to stress and drug-cue exposure (Smith et al., 2023). Also, stress and stimulants cause maladaptive decision-making through epigenetic changes in the dorsal striatum (Murphy and Heller, 2022). Therefore, stress influences many substance use aspects, from consumption maintenance through neurotransmitter systems modulation to a contextual association that elicits drug use resumption (Nazeri et al., 2017; Goldfarb and Sinha, 2018; Mukhara et al., 2018).

### 1.2 The early-life stress implications for SUD

The ELS is among the major risk factors for psychiatric disorders development—for example, substance use, mood, anxiety, and posttraumatic stress disorders are clinical outcomes of severe ELS (Berhe et al., 2022). Neglect, trauma, family dysfunction, or abuse in general has about 3.6 million annual reports, and ~702,000 children are confirmed victims of abuse or neglect (Forster et al., 2018). ELS has been associated with a higher risk of mood disorders (Forster et al., 2018; Andersen, 2019) and SUD (Goodwin et al., 2004; Kirsch and Lippard, 2022). Substance abuse can emerge to alleviate suffering, anxiety, and childhood trauma, resulting in substance dependence to manage their emotional experiences, establishing a vicious cycle (Bushnell et al., 2019).

The proper development of the CNS requires essential cellular processes and must be fine-tuned to ensure its adequate formation (Andersen, 2003). It is known that substance use alters the structure and function of serotonergic and dopaminergic neurons during adolescence, making the developing brain highly susceptible to the neurotoxic effects of drug exposure (Squeglia et al., 2012; Pfefferbaum et al., 2018). For example, neural activation and volume of cortical areas in adolescents predict alcohol consumption and alcohol-related problems (Norman et al., 2011; Cheetham et al., 2014). On the other hand, ELS can affect neurons and glial cells during neurodevelopment (Schafer and Stevens, 2015; Allen and Lyons, 2018; Johnson and Kaffman, 2018; Li and Barres, 2018), including structures and components of the reward system (Lukkes et al., 2009; Hanson et al., 2021; Moustafa et al., 2021). Indeed, there is an important link between ELS and SUD development through adolescent substance use (Kirsch and Lippard, 2022). Rodent models show that different stressors during adolescence or the corresponding pre-adolescence period increase drug consumption during adulthood (Kosten et al., 2000, 2004; Baarendse et al., 2014; Garcia-Pardo et al., 2015). Maternal separation (MS), an ELS closer to the time of birth, has also been shown to increase self-administered alcohol drinking and morphine preference during adulthood (Jaworski et al., 2005; Vazquez et al., 2005; Michaels and Holtzman, 2008; Gondre-Lewis et al., 2016; Lewis et al., 2016). Previous studies reported arginine vasopressin gene expression changes and HPA axis activation after MS (Murgatroyd and Spengler, 2011; de Almeida Magalhaes et al., 2018). The HPA axis's ability to influence substance use seems to be so important that it has been placed as a potential target to assess the probability of relapse in cocaine-dependent individuals (Sinha et al., 2006). The ELS occurring in a range from weaning to early adulthood can affect substance use (McCool and Chappell, 2009; Lopez et al., 2011), suggesting that any period during early life is sensitive to stress effects with crucial implications for SUD development.

### 1.3 SUD and stress at cellular level

Stress occurs not only at psychological and physiological levels but also at a cellular level, e.g., oxidative stress and neuroinflammation. Recent findings highlight oxidative stress and inflammation as pivotal factors in drug-induced disruption of brain homeostasis (Berrios-Carcamo et al., 2020). For example, research in mice has demonstrated that the administration of indomethacin, a potent anti-inflammatory agent, reduced methamphetamine-induced neuroinflammation (Goncalves et al., 2008) and prolonged use of various addictive substances elevates inflammatory responses in the periphery and central nervous system (CNS; Cahill and Taylor, 2017; Leclercq et al., 2017; Hofford et al., 2019; Kohno et al., 2019). This situation could initiate an inflammatory response through increased microglial and astrocytic reactivity (Kraft and Harry, 2011; Clark et al., 2013; Colombo and Farina, 2016). Moreover, increased microglial and astrocytic reactivity has been observed in response to amphetamines (Zhang et al., 2015), cocaine (Periyasamy et al., 2018), ethanol, nicotine (Alfonso-Loeches et al., 2010; Quintanilla et al., 2018, 2019), opioids (Wang et al., 2012), and cannabinoids (Cutando et al., 2013; Zamberletti et al., 2015). These cells can sense cellular environmental alterations and trigger inflammatory responses through pattern recognition receptors, including Toll-like receptors (TLRs; Kraft and Harry, 2011; Fischer and Maier, 2015). Microglia respond to pro-inflammatory signals by altering their reactivity and gene expression, leading to elevated production of oxidative enzymes like NADPH oxidase (NOX) and inducible nitric oxide synthase (iNOS). This response increases the generation of reactive oxygen species (ROS) and reactive nitrogen species (RNS; Block et al., 2007). Cocaine and opioids induce TNF-α, IL-1β, and IL-6 release through microglial and astrocytic TLR4 activation, and IL-1β, IL-6, IL-18, IL-33, MCP-1, and TNF-α production via NF-κB and NLRP3 inflammasome pathways (Hutchinson et al., 2010; Crews et al., 2013; Northcutt et al., 2015; Pan et al., 2016; Bayazit et al., 2017; Eidson et al., 2017; Berrios-Carcamo et al., 2020; Figure 1B).

Studies in animal models have shown that chronic alcohol use increases pro-inflammatory cytokines, inhibits neurogenesis, and induces long-term behavioral changes (Nixon and Crews, 2002; Pascual et al., 2007). Furthermore, excessive DA released in response to methamphetamine undergoes oxidation, leading to the formation of toxic quinones. This process triggers oxidative stress, causes mitochondrial dysfunction, and damages presynaptic membranes by generating free radicals like superoxide and hydrogen peroxide (Shah et al., 2012). The cause of oxidative stress in the brain may be due to excessive production of free radicals, decreased activity of antioxidant enzymes, or decreased concentration of reducing factors (Lin and Beal, 2006; Kaminski et al., 2024), where ROS and RNS, for example, exert toxic effects on the CNS cellular components, resulting in neuronal death (Berg et al., 2004). Several studies showed that SUDs and oxidative stress are linked since the presence of one correlates with the other's development (Cunha-Oliveira et al., 2010; Zahmatkesh et al., 2017; Kaminski et al., 2024). Cannabis smoke exposure increases oxidative stress, like tobacco's effect (Aguiar et al., 2019), leading to increased ceruloplasmin and lipid hydroperoxides and decreased free thiol (Bayazit et al., 2020). Indeed, tetrahydrocannabinol (THC), a psychoactive substance found in cannabis, increases lipoperoxidation and reduces superoxide dismutase (SOD) enzyme activity in brain tissue (Kopjar et al., 2019). Exposure to amphetamines damages the mitochondrial membrane and oxidates lipids and proteins through increased ROS production (Brown and Yamamoto, 2003; Fitzmaurice et al., 2006; Perfeito et al., 2013; Basmadjian et al., 2021). Cocaine depletes reduced glutathione (GSH) in the heart and liver (Graziani et al., 2016), decreases catalase activity in the striatum and mPFC (Macedo et al., 2005), and glutathione peroxidase and GSH reduction in HPC (Mahoney, 2019). Finally, studies indicate that heroin increases ROS production and oxidative damage to proteins and lipids in the brain and liver (Graziani et al., 2016), decreases SOD, CAT, and GPx activity, and GSH/glutathione disulfide ratio reduction (Cemek et al., 2011; Zahmatkesh et al., 2017; Salarian et al., 2018; Tomek et al., 2019).

### 1.4 Genetic hallmarks of SUD

**Figure 2:**
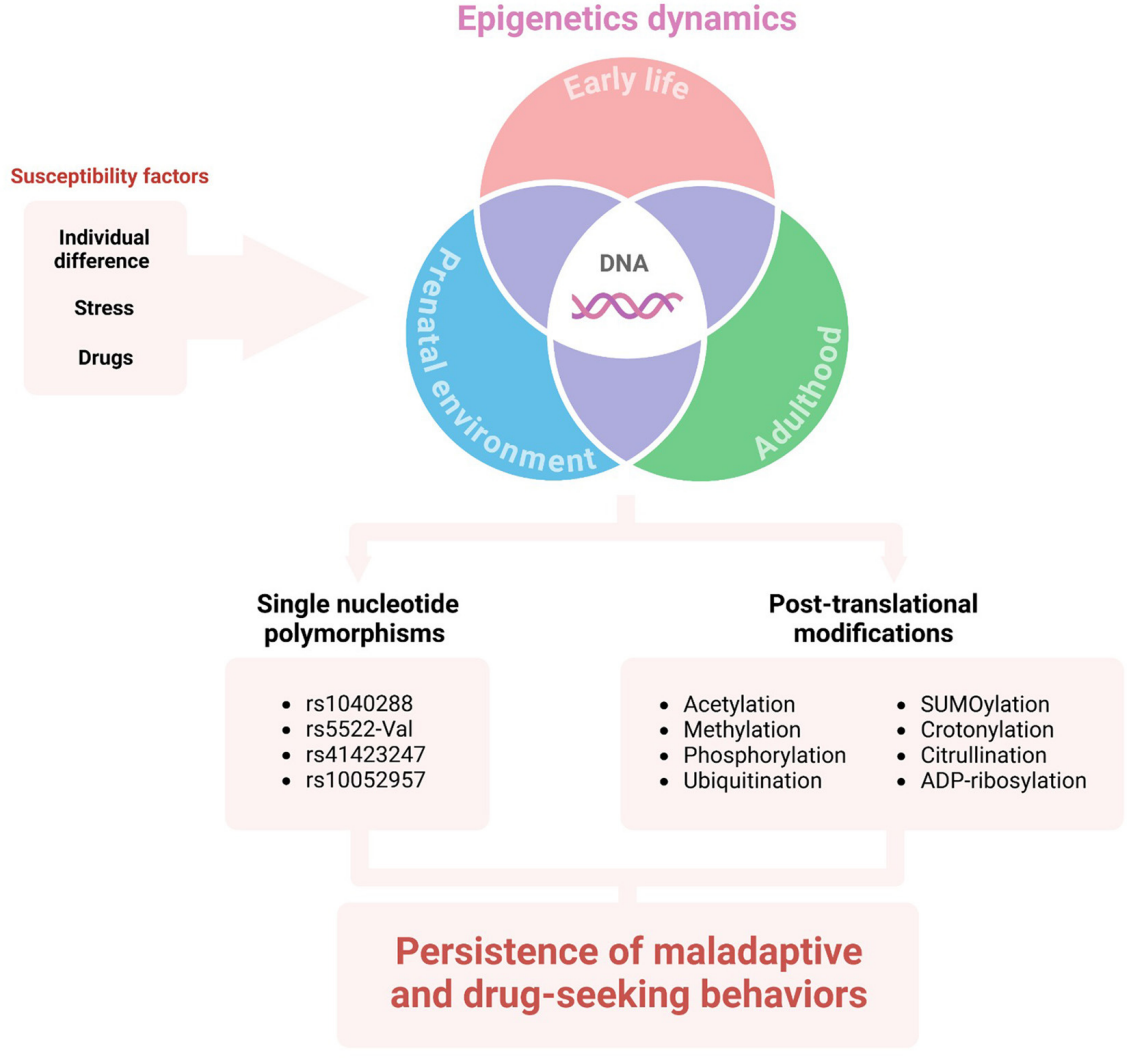
The influence of DNA epigenetics on vulnerability to SUD. Individual differences (e.g., environment, habits, life history, genetic background), as well as stress and/or drug exposure may lead to epigenetic alterations in different life stages. The single nucleotide polymorphisms and post-translational modifications of HPA-axis genes or mediators can sustain maladaptive behavior and substance use disorder.

Some fundamental questions, such as “Why are some individuals more vulnerable to SUDs than others?” and “Does stress influence individual vulnerability?” remain unanswered. A possible mechanism for individual vulnerability to stress-induced substance use is through epigenetic modulation (Figure 2). There is substantial evidence for ELS-induced epigenetic changes influencing substance use in adulthood (Provencal and Binder, 2015). Also, ELS seems to induce dense DNA methylation of the GR gene (*NR3C1*), which correlates with major depressive disorder (Holmes et al., 2019). Interestingly, substance use also induces epigenetic changes, such as post-translational modifications, acetylation, methylation, phosphorylation, ubiquitination, SUMOylation, crotonylation, citrullination, and ADP-ribosylation, as well as methylation of the DNA itself (Nestler, 2014; Walker and Nestler, 2018). Several miRNAs are regulated after drug exposure (Doura and Unterwald, 2016), with the expression of some in striatum neurons altering drug-related behaviors (Hollander et al., 2010; Chandrasekar and Dreyer, 2011; Quinn et al., 2015).

Additionally, it is well known that minor genetic variations between the population correlate with variations in disorder development risk. Single nucleotide polymorphisms (SNPs) in genes associated with the HPA axis can modify the risk for drug abuse and abstinence symptoms. For example, the *NR3C2* gene located in the 4q31.1v chromosome encodes the MR. The rs1040288 SNP results in a displacement of G to C nucleotide in an intronic region of the gene and has been identified as a risk factor for cocaine and heroin abuse in a non-population-specific manner (Levran et al., 2014). Regarding ELS, there is an association between childhood physical neglect and the SNP rs5522-Val allele modulating crack/cocaine abuse (Rovaris et al., 2015). The rs5522 SNP consists of an A/G transition in an exonic region of the gene, which results in a substitution of isoleucine to a valine.

On the other hand, GR is encoded by the *NR3C1* gene located at the 5q31-32 chromosome. Atypical GR sensitivity underpins the pathophysiology of drug abuse, continuation, and relapse. The rs41423247 SNP consists of a displacement of G to C nucleotide in an intronic region of the gene. This SNP homozygous mutation has been associated with depression (Peng et al., 2018). Its minor allele C is a risk factor for higher depressive symptoms during early abstinence from crack/cocaine abuse, while the CC genotype appears to correlate with late abstinence (Rovaris et al., 2016). The rs41423247 minor allele C and rs10052957 minor allele G (an SNP that results in displacement of A to G) have been associated with an increased risk for cocaine abuse and a higher burden of depression when combined in a haplotype (Schote et al., 2019).

## 2 Discussion and conclusions

This mini-review explores how substance use alters brain circuits involved in reward processing and stress response (the “anti-reward” system), linking these changes to the three-stage model of SUD and their anatomical and endocrine features. It integrates cellular stress, which is closely tied to SUD. Depending on its nature, intensity, and duration, stress impacts HPA axis modulation, brain plasticity, and cellular processes (Albernaz-Mariano et al., 2025). Thus, ELS was highlighted due to its strong translational body of evidence and association with blunted stress responses and increased SUD risk (initiation, maintenance, relapse), mediated by ELS-induced reward and stress pathways changes. Finally, individual vulnerability to SUD was examined through (epi)genetics, emphasizing how drug use and life experiences can alter gene expression and increase SUD risk in susceptible individuals.

In conclusion, substance use can disrupt major brain circuits and neuroendocrine systems, resulting in altered behavioral responses to reward and stress. Furthermore, exposure to stress, particularly early-life stress (ELS), may increase susceptibility to substance use disorder (SUD) during adolescence and adulthood. Cellular stress induced by either stress or SUD plays a significant role in this process, offering potential therapeutic targets. Additionally, genetic factors may provide a means to identify at-risk individuals, enabling early intervention and prevention of SUD development.
